# Left ventricular dysfunction, adverse myocardial and aortic remodeling in patients with tetralogy of Fallot without symptoms of heart failure after surgical repair

**DOI:** 10.1186/1532-429X-17-S1-W25

**Published:** 2015-02-03

**Authors:** Ana C Andrade, Michael Jerosch-Herold, Inga Voges, Minh H Pham, Ravi V Shah, Christopher Hart, Philip Wegner, Hans-Heiner Kramer, Carsten Rickers

**Affiliations:** 1 Cardiology, Heart Institute, São Paulo, Brazil; 2 Brigham and Women's Hospital, Harvard Medical School, Boston, MA, USA; 3 Klinik für angeborene Herzfehler und Kinderkardiologie, Universitätsklinikum Schleswig-Holstein, Kiel, Germany

## Background

Repair of tetralogy of Fallot (ToF) frequently has long-term sequelae of right ventricle (RV) dysfunction, which may also lead to left ventricular (LV) dysfunction due to unfavorable RV coupling. We hypothesized that ToF can lead to adverse left heart remodeling, including LV myocardial extracellular matrix expansion, hallmarks of a cardiomyopathic process.

## Methods

Cardiac magnetic resonance (CMR) was performed at 3.0 Tesla in 109 asymptomatic ToF patients, (age 19.3±12.8 years) after surgical correction (16.6±10.0 years post operation), and 64 age-matched controls. Parameters of LV and left atrial (LA) function and aortic distensibility were obtained from cine CMR. In a subgroup (n=50), T1 mapping was used to determine the myocardial extracellular volume fraction (ECV) as an index of diffuse myocardial fibrosis.

## Results

Compared to control subjects, ToF patients had a lower LV ejection fraction (EF) (50.7 versus 59.0±5.4; p<0.01), despite a similar RV EF (p=0.34). ToF showed a lower LV mass index, lower LV mass-to-volume ratio, and lower aortic distensibility. LA passive volume and LA total ejection fraction were decreased, and associated with age (p<0.01), suggesting an early onset of diastolic dysfunction. ECV was elevated in ToF (0.32±0.05 versus 0.26±0.01 in controls (p<0.01), more so in females (p<0.05), and was inversely associated with LV mass index (p<0.05).

## Conclusions

During long-term follow-up after repair of ToF, asymptomatic patients show alterations in LV geometry, function, tissue structure, and aortic distensibility. These findings indicate an early adverse cardiovascular phenotype and underscore the need for life-long follow-ups.

## Funding

This work was supported by a Postdoctoral Fellowship grant from Coordination of Improvement of Higher Education Personnel (CAPES), Brazil.

